# Development and validation of a nomogram prognostic model for esophageal cancer patients with oligometastases

**DOI:** 10.1038/s41598-020-68160-6

**Published:** 2020-07-09

**Authors:** Butuo Li, Ruiqing Wang, Ting Zhang, Xiubin Sun, Chao Jiang, Wanlong Li, Bing Zou, Peng Xie, Xue Meng, Xindong Sun, Linlin Wang, Jinming Yu

**Affiliations:** 1 National Clinical Research Center for Cancer, Key Laboratory of Cancer Prevention and Therapy, Tianjin Medical University Cancer Institute and Hospital, Tianjin, 300060 China; 2 Department of Radiation Oncology, Tianjin Medical University, Tianjin, 300070 China; 3 Department of Radiation Oncology, Shandong Cancer Hospital and Institute, Shandong First Medical University and Shandong Academy of Medical Science, Jiyan Road 440, Jinan, 250117 Shandong Province China; 4 Department of Breast Surgery, Linyi People’s Hospital, Linyi, 276000 Shandong Province China; 5 Jinan Infectious Disease Hospital Affiliated To Shandong University, Jinan, 250012 Shandong Province China; 6 Department of Biostatistics, School of Public Health, Shandong University, Jinan, 250012 Shandong Province China; 7 Department of Otorhinolaryngology and Head and Neck Surgery, Provincial Hospital Affiliated To Shandong University, Jinan, 250000 Shandong Province China

**Keywords:** Cancer therapy, Oesophageal cancer, Risk factors

## Abstract

Platinum-based chemotherapy is recommended as the standard treatment for metastatic esophageal cancer (EC) patients; however, the outcome is poor. Oligometastasis is less aggressive and has limited growth potential. However, the prognostic factors for EC patients with oligometastases was largely unknown. Thus, we intend to determine the prognostic factors, and develop and validate nomograms for prediction of survival for EC patients with oligometastases. In this study, characteristics of 273 oligometastatic EC patients were analyzed using univariate and multivariate Cox models to determine the independent prognostic factors for progression-free survival (PFS) and overall survival (OS). The result showed that history of alcohol consumption, longer tumor, no local radiotherapy for EC, and no local treatment for metastases were independent factors for PFS. Sex, esophageal fistula, number of metastatic organs, and local radiotherapy for EC were independent prognostic factors for OS. On the basis of Cox models, the respective nomogram for prediction of PFS and OS was established with the corrected concordance index of 0.739 and 0.696 after internal cross-validation. In conclusion, local treatment for metastases and local radiotherapy for EC were demonstrated to be beneficial for oligometastatic EC patients, and the validated nomograms are valuable in prognosis prediction and could guide individualized management for these patients.

## Introduction

Esophageal cancer (EC) is the seventh most common cancer and sixth leading cause of cancer-related deaths globally, which is characterized by the regional diversity of incidence rate and pathological patterns^1^. Platinum-based chemotherapy is recommended as the standard treatment for metastatic EC patients; however, the outcome is poor with a response of 20–50%^2^ and 5-year survival < 5%^3^. It is therefore necessary for the risk assessment of disease progression and death, which may result in individualized therapies and improvement of patient survival.

Within the population with cancer metastasis, oligometastases, firstly proposed by Hellman and Weichselbaum^4^, are characterized by the presence of fewer than five metastases^5^. Compared with the strongly invasive and metastatic properties of polymetastatic disease, oligometastatic disease is less aggressive and has limited growth potential^6^. In non-small-cell lung cancer, oligometastastic patients have better prognosis than polymetastatic cases^7^. However, only a small number of studies have been published to address the prognosis of patients with EC regarding the number of metastases. Prognostic factors for metastatic EC patients have been investigated, and better performance, limited metastases, and aggressive primary tumor radiotherapy were found to be associated with improved survival^8–10^. However, the prognostic factors for EC patients with oligometastases were largely unknown.

An effective tool is crucial for the differentiation of high-risk patients, who will obtain clinical benefit from intensive therapy. However, there are currently no such tools available for evaluation of prognosis of EC patients with oligometastases. The nomogram, a simple graphic representation of a statistical prediction model, has successfully been developed in lung^11^ and breast^12^ cancer and others^13^. Importantly, it has been validated to be superior to the traditional TNM staging systems in the prognosis prediction of cancer patients. A nomogram based on the clinical factors and treatment strategies may be promising in the quantitative evaluation of survival in EC patients with oligometastases.

We therefore conducted this study to explore the prognostic factors with regard to progression-free survival (PFS) and overall survival (OS) in EC patients with oligometastases, and to develop practical nomogram models for their prognosis prediction.

## Results

### Patient characteristics

Among 576 patients with metastatic EC, 273 (47.4%) of them had oligometastases and 303 (52.6%) patients had polymetastatic EC. 273 oligometastatic EC patients were enrolled in this study, and 152 patients died and 229 patients developed disease progression within a median follow-up of 14.2 mo. Among 229 patients with disease progression, 121 patients had enlargement of original lesions, 59 patients had newly-occurred metastases, and 49 patients had both newly-occurred metastases and enlargement of original lesions.

Patient, tumor and treatment characteristics are shown in Table 1. The median age of all patients was 61 years (range 50–86 years), and male-to-female ratio was 224: 49. 96.3% of patients had Eastern Cooperative Oncology Group (ECOG) of 0–1. Seventeen patients experienced esophageal fistula due to tumor development or treatment. In terms of tumor characteristics, most of the tumors were located in the thoracic esophagus and 91.2% was squamous cell cancer. One hundred and forty-one of 170 patients received definitive dose radiotherapy (≥ 5,040 cGy) of the primary esophageal tumor, while others received palliative dose radiotherapy. In addition, 148 patients received local treatment for metastases, and 142 among them received radiotherapy, 6 received interventional chemoembolization.

**Table 1:** Patients, tumor and treatment characteristics.

Variables	No. (%)
**Patient characteristics**	
Age	
≤ 61	142 (52%)
> 61	131 (48%)
Sex	
Male	224 (82.1%)
Female	49 (17.9%)
ECOG performance status	
0–1	263 (96.3%)
2–3	10 (3.7%)
Smoking history	
No	116 (42.5%)
Yes	157 (57.5%)
Smoking index	
< 600	170 (62.3%)
≥ 600	103 (37.7%)
History of alcohol consumption	
No	121 (44.3%)
Yes	152 (55.7%)
Esophageal fistula	
No	256 (93.8%)
Yes	17 (6.2%)
**Tumor characteristics**	
Location	
Cervical	6 (2.2%)
Upper thoracic	58 (21.3%)
Middle thoracic	94 (34.4%)
Lower thoracic	103 (37.7%)
Synchronous	12 (4.4%)
Pathological type	
Squamous	249 (91.2%)
Adenocarcinoma	7 (2.6%)
Small cell	17 (6.2%)
Longitudinal length	
T stage	
T1–T3	203 (74.4%)
T4	66 (24.2%)
Unknown	4 (1.6%)
N stage	
N0–N1	123 (45.1%)
N2–N3	150 (54.9%)
Number of metastases	
1	131 (48%)
2	98 (35.9%)
3	26 (9.5%)
4	11 (4%)
5	7 (2.6%)
Number of metastatic organs	
1	170 (62.3%)
2	91 (33.3%)
3	12 (4.4%)
Distant lymph node	
0–1	188 (68.9%)
> 1	85 (31.1%)
Liver metastasis	
No	244 (89.4%)
Yes	29 (10.6%)
Lung metastasis	
No	244 (89.4%)
Yes	29 (10.6%)
Bone metastasis	
No	258 (94.5%)
Yes	15 (5.5%)
**Treatment characteristics**	
Treatment protocol	
Single chemotherapy	94 (34.4%)
Concurrent chemoradiotherapy	59 (21.6%)
Sequential chemoradiotherapy	91 (33.3%)
Single radiotherapy	18 (6.6%)
Surgery + adjuvant therapy	11 (4%)
Local radiotherapy for EC	
No	103 (37.7%)
Yes	170 (62.3%)
Local treatment for metastases	
No	125 (45.8%)
Partial metastases	18 (6.6%)
All metastases	130 (47.6%)

EC, esophageal cancer.

### Survival analysis

The median PFS was 7.9 mo (95% confidence interval (CI) 7.0–8.8 mo), and PFS rate was 63.6% and 25.7% at 6 and 12 mo, respectively. Median OS was 18.4 mo (95% CI 16.1–20.8 mo), and the survival rate was 68.2% at 12 mo and 38.7% at 24 mo. The results of univariate and multivariate Cox analyses are shown in Tables 2 and 3, respectively.

**Table 2:** Univariate and multivariate Cox models for PFS.

Variables	Uni HR	95% CI	*P* value	Multi HR	95% CI	*P* value
**Patient characteristics**						
Age						
≤ 61	1					
> 60	0.73	0.56–0.95	0.02			
Sex						
Male	1					
Female	0.69	0.49–0.98	0.04			
ECOG performance status						
0–1	1					
2–3	1.97	1.04–3.73	0.038			
Smoking index						
< 600	1					
≥ 600	1.40	1.07–1.83	0.014			
History of alcohol consumption						
No	1			1		
Yes	1.47	1.13–1.91	0.004	1.37	1.03–1.84	0.033
Esophageal fistula						
No	1					
Yes	1.51	0.91–2.51	0.11			
**Tumor characteristics**						
Location						
Cervical	1					
Upper thoracic	0.95	0.38–2.40				
Middle thoracic	1.27	0.52–3.14				
Lower thoracic	1.22	0.49–3.00				
Synchronous	1.77	0.62–5.03	0.33			
Pathological type						
Squamous	1					
Adenocarcinoma	0.67	0.28–1.62				
Small cell	0.76	0.44–1.31	0.42			
Longitudinal length	1.08	1.02–1.13	0.06	1.09	1.04–1.15	0.001
T stage						
T1–T3	1					
T4	1.15	0.85–1.57	0.37			
N stage						
N0–N1	1					
N2–N3	1.35	1.04–1.75	0.027			
Number of metastases						
1	1					
2	1.33	1.0–1.78				
3	1.08	0.67–1.74				
4	2.53	1.36–4.74				
5	3.02	1.31–6.95	0.004			
Number of metastatic organs						
1	1					
2	1.35	1.02–1.78				
3	1.73	0.93–3.20	0.041			
Distant lymph node						
0–1	1					
> 1	1.14	0.86–1.51	0.33			
Liver metastasis						
No	1					
Yes	1.20	0.80–1.79	0.38			
Lung metastasis						
No	1					
Yes	1.63	1.07–2.48	0.022			
Bone metastasis						
No	1					
Yes	1.32	0.75–2.32	0.33			
**Treatment characteristics**						
Treatment protocol						
Single chemotherapy	1					
Concurrent chemoradiotherapy	0.35	0.24–0.50				
Sequential chemoradiotherapy	0.33	0.24–0.45				
Single radiotherapy	0.33	0.20–0.58				
Surgery + adjuvant therapy	0.55	0.28–1.07	< 0.001			
Local radiotherapy for EC						
No	1			1		
Yes	0.31	0.24–0.41	< 0.001	0.37	0.24–0.56	< 0.001
Local treatment for metastases						
No	1			1		
Partial metastases	0.43	0.25–0.76		0.56	0.28–1.11	
All metastases	0.40	0.30–0.53	0.00	0.63	0.42–0.96	0.032

EC, esophageal cancer; PFS, progression free survival; HR, hazard ratio; CI, confidence interval.

**Table 3:** Univariate and multivariate Cox models for OS.

Variables	Uni HR	95% CI	*P* value	Multi HR	95% CI	*P* value
**Patient characteristic**						
Age						
≤ 61	1					
> 60	0.72	0.52–1.00	0.046			
Sex						
Male	1			1		
Female	0.57	0.34–0.94	0.027	0.48	0.27–0.88	0.016
Smoking index						
< 600	1					
≥ 600	1.42	1.03–1.96	0.034			
ECOG performance status						
0–1	1					
2–3	1.38	0.51–3.74	0.53			
History of alcohol consumption						
No	1					
Yes	1.37	0.99–1.89	0.06			
Esophageal fistula						
No	1			1		
Yes	2.70	1.58–4.62	< 0.001	2.5	1.35–4.64	0.004
**Tumor characteristics**						
Location						
Cervical	1					
Upper thoracic	3.64	0.50–26.6				
Middle thoracic	3.73	0.52–27.0				
Lower thoracic	3.76	0.52–27.1				
Synchronous	8.34	1.05–66.0	0.12			
Pathological type						
Squamous	1					
Adenocarcinoma	0.83	0.31–2.25				
Small cell	1.08	0.60–1.96	0.90			
Longitudinal length	1.07	1.00–1.14	0.052			
T stage						
T1–T3	1					
T4	1.11	0.76–1.62	0.59			
N stage						
N0–N1	1					
N2–N3	1.25	0.91–1.72	0.18			
Number of metastases						
1	1					
2	1.60	1.13–2.28				
3	1.29	0.71–2.34				
4	2.55	1.27–5.12				
5	2.82	0.88–9.05	0.013			
Number of metastatic organs						
1	1			1		
2	1.59	1.13–2.25		1.74	1.18–2.56	
3	2.11	1.02–4.35	0.009	2.00	1.25–7.21	0.001
Distant lymph node						
0–1	1					
> 1	1.54	1.09–2.17	0.014			
Liver metastasis						
No	1					
Yes	0.96	0.55–1.68	0.89			
Lung metastasis						
No	1					
Yes	1.66	1.04–2.66	0.035			
Bone metastasis						
No	1					
Yes	1.14	0.58–2.24	0.70			
**Treatment characteristics**						
Treatment protocol						
Single chemotherapy	1					
Concurrent chemoradiotherapy	0.53	0.34–0.83				
Sequential chemoradiotherapy	0.50	0.34–0.74				
Single radiotherapy	0.82	0.44–1.54				
Surgery + adjuvant therapy	0.69	0.31–1.54	0.006			
Local radiotherapy for EC						
No	1			1		
Yes	0.51	0.37–0.71	< 0.001	0.15	0.23–0.93	0.041
Local treatment for metastases						
No	1					
Partial metastases	0.44	0.20–0.96				
All metastases	0.52	0.37–0.72	< 0.001			

EC, esophageal cancer; OS, overall survival; HR, hazard ratio; CI, confidence interval.

On univariate analyses, ≤ 61 male patients with higher smoking index or history of alcohol consumption were found to have inferior PFS and OS, and poor ECOG performance status was associated with inferior PFS but not OS. In the field of tumor characteristics, the longitudinal length, N stage, number of metastases and metastatic organs, and lung metastasis were found to be significant factors of disease progression; and the longitudinal length, number of metastases and metastatic organs, distant lymph node and lung metastasis were found to be predictive factors for death. Besides, treatment protocol, local radiotherapy for EC and local treatment for metastases were also found to be associated with the PFS and OS.

**Figure 1:**
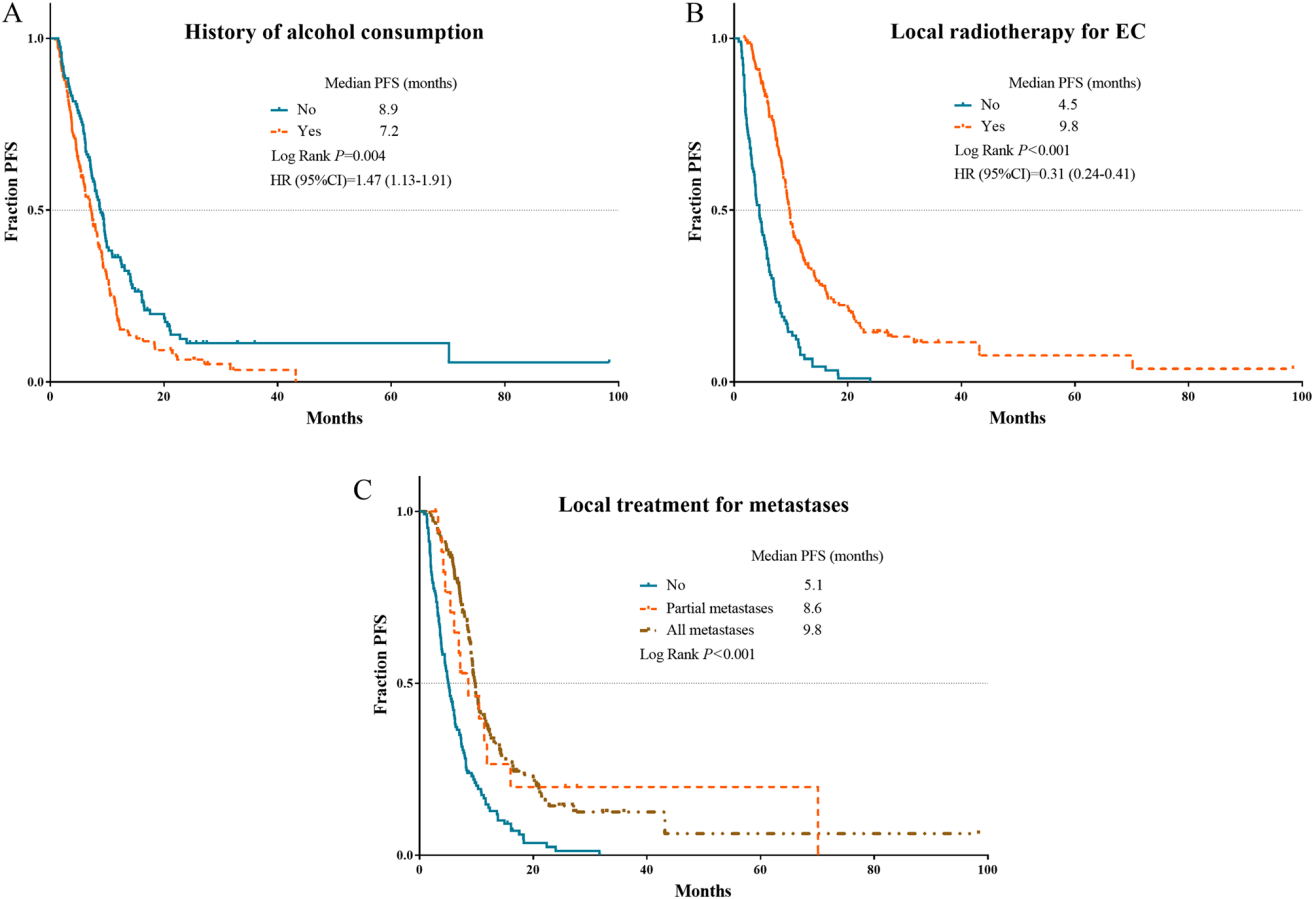
Kaplan Meier survival plots of independent risk factors for PFS in oligometastatic EC patients. (**A**) History of alcohol consumption; (**B**) local radiotherapy for EC; (**C**) local treatment for metastases. PFS, progression free survival; EC, esophageal cancer.

**Figure 2:**
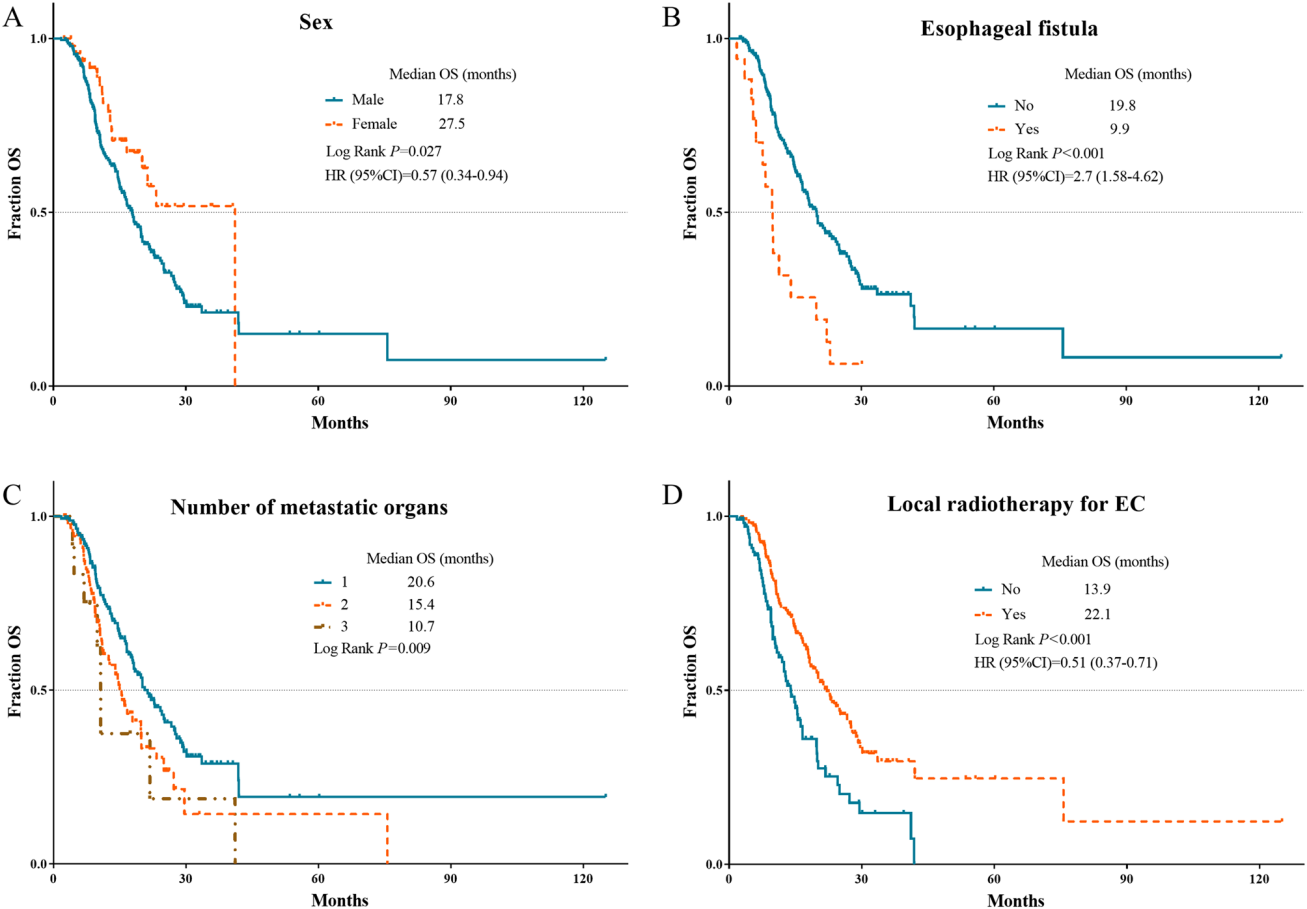
Kaplan Meier survival plots of independent risk factors for OS in oligometastatic EC patients. (**A**) Sex; (**B**) esophageal fistula; (**C**) number of metastatic organs; (**D**) local radiotherapy for EC. OS, overall survival; EC, esophageal cancer.

Multivariate analyses of PFS demonstrated that history of alcohol consumption, longer tumor, lack of local radiotherapy for EC and lack of local treatment for metastases were independent factors for disease progression. Sex, esophageal fistula, number of metastatic organs, and local radiotherapy for EC were independent prognostic factors for OS. However, number of metastasis has lost the significance in multivariate analyses of PFS and OS. The independent risk factors for PFS and OS were validated in survival curves using Kaplan–Meier analyses (Figs. 1, 2).

### Prognostic nomogram for PFS and OS

**Figure 3:**
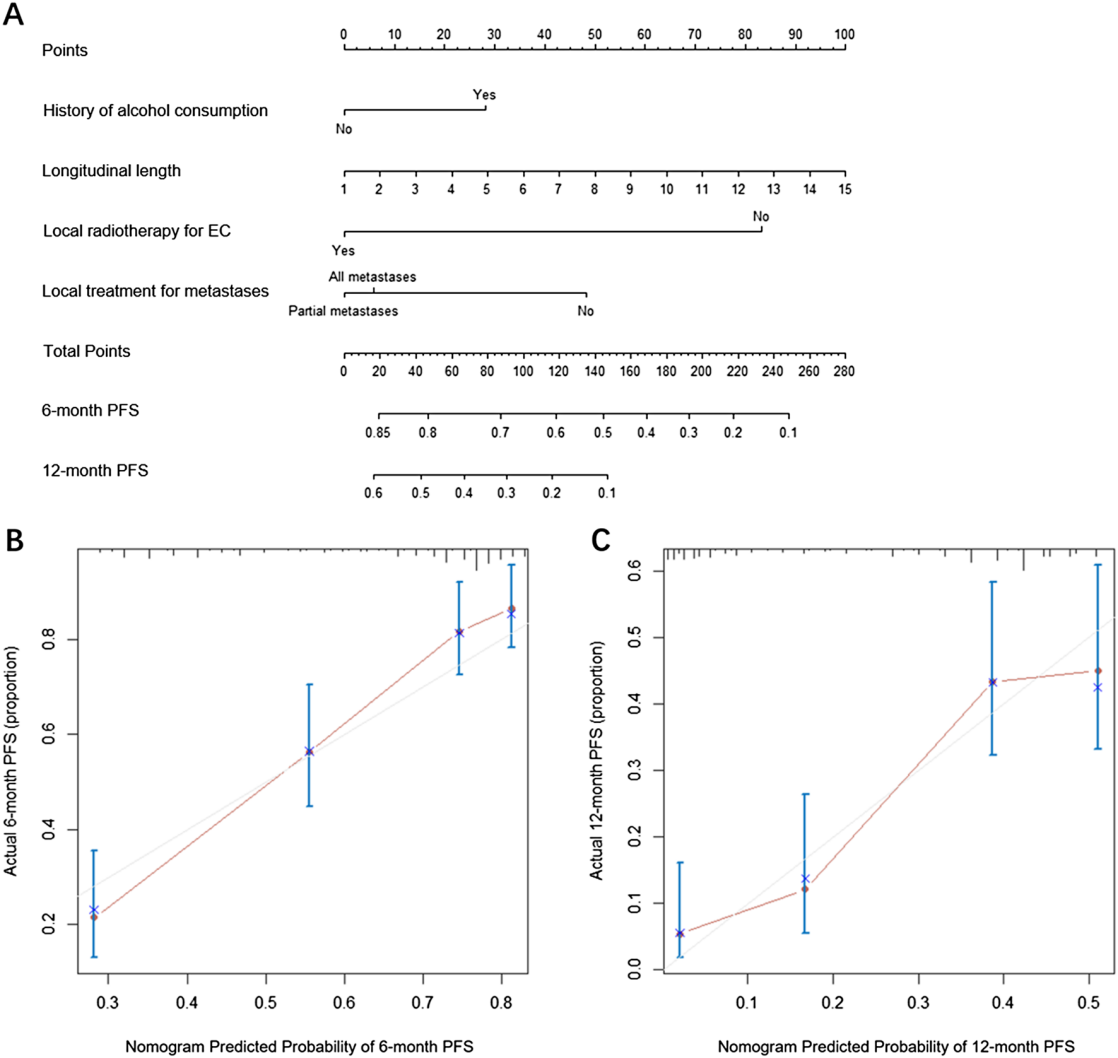
Nomogram and calibration curve for predicting PFS at 6- and 12-month for oligometastatic EC patients. (**A**) Nomogram; The nomogram is used by adding the point identified on the points scale of each variable. The sum of points is located on the Total Points axis, and a line is drawn down to indicate the 6- and 12-month PFS. (**B**) Calibration curve for 6-month PFS; (**C**) Calibration curve for 12-month PFS. PFS, progression free survival; EC, esophageal cancer.

**Figure 4:**
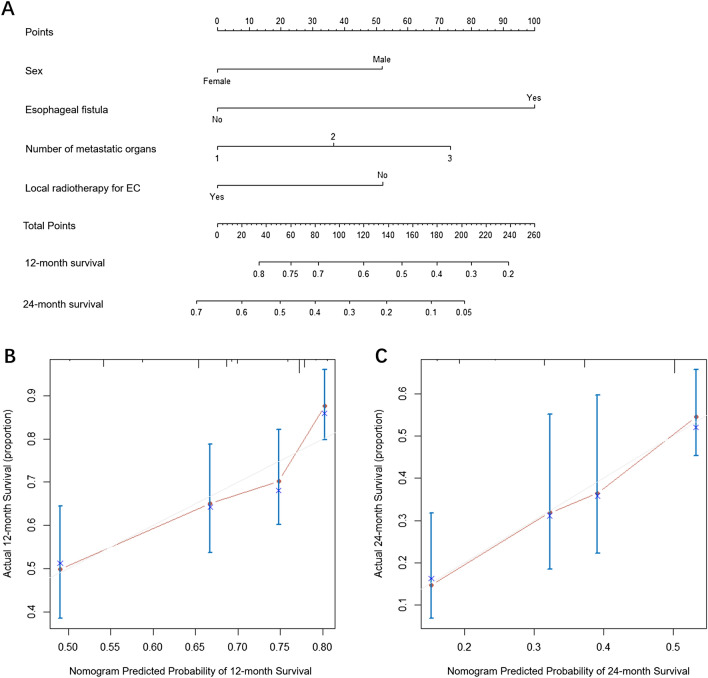
Nomogram and calibration curve for predicting OS at 12- and 24-month for oligometastatic EC patients. (**A**) Nomogram; The nomogram is used by adding the point identified on the points scale of each variable. The sum of points is located on the Total Points axis, and a line is drawn down to indicate the 12- and 24-month survival. (**B**) Calibration curve for 12-month OS; (**C**) calibration curve for 24-month OS. OS, overall survival; EC, esophageal cancer.

The prognostic nomogram for PFS and OS (Figs. 3A, 4A) was established by integrating independent factors for PFS and OS, respectively. The nomogram of PFS assigned points based on history of alcohol consumption, longitudinal length of tumor, local radiotherapy for EC and local treatment for metastases. Outcomes were reported as 6- and 12-months PFS. The nomogram of OS included sex, esophageal fistula, number of metastatic organs, and local radiotherapy for EC, and outcomes were reported as 12- and 24-month OS. The uncorrected concordance index (C-index) was 0.75 for PFS prediction and 0.734 for OS prediction, and the corrected C-index generated by internal cross-validation was 0.739 and 0.696, respectively. The calibration plot for the probability of PFS at 6 or 12 mo (Fig. 3B, C), and survival at 12 or 24 mo (Fig. 4B, C) illustrated the optimal agreement between the actual observation and the prediction of PFS and OS by the nomogram.

**Figure 5:**
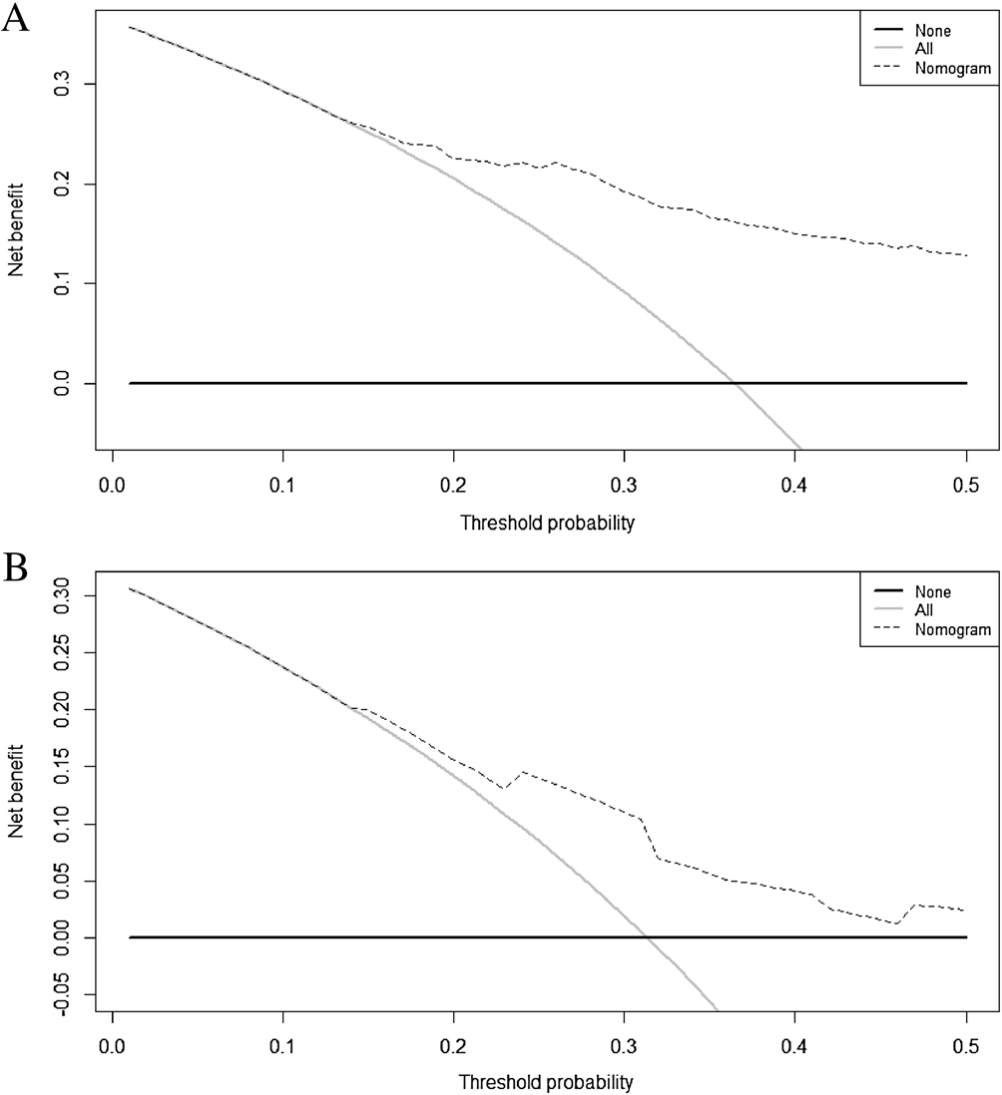
Decision curve analysis of nomogram model for predicting PFS and OS of oligometastatic EC patients. The dash line represents the net benefit of nomogram model. (**A**) Decision curve analysis of nomogram models for predicting 6-month PFS of oligometastatic EC patients. The gray and black lines represent that no patients and all patients would progress, respectively. (**B**) Decision curve analysis of nomogram models for predicting 12-month survival of oligometastatic EC patients. The gray and black lines represent that no patients and all patients would die, respectively. PFS, progression free survival; OS, overall survival; EC, esophageal cancer.

Decision curve analysis (DCA) were performed in order to determine the reliability of results of nomograms-based predictions for different decision thresholds. Decision curves for the prediction of progression after 6 months and death after 12 months are shown in Fig. 5A, B, respectively. The displayed DCA showed a favorable prediction of the nomogram for disease progression and death.

## Discussion

Oligometastatic EC has favorable prognosis due to its indolent property, when compared to polymetastatic EC^8,9^. However, the prognostic factors for oligometastatic EC patients were still unclear. Our study, for the first time, performed survival analyses for individualized prediction of EC patients with oligometastases, and developed and validated nomograms to quantitatively evaluate survival. The nomograms permitted integration of patient, tumor and treatment characteristics, and provided patient-specific estimates of OS and PFS, which could be used for the prognosis prediction of EC patients with oligometastases.

In previous studies, history of alcohol consumption was a risk factor and led to an adverse outcome for esophageal squamous cell carcinoma (ESCC) patients^14,15^. Similarly, history of alcohol consumption was found to be an independent prognostic factor for PFS in oligometastatic EC patients in the present study. Cumulative exposure to alcohol may cause more aggressive phenotype, then result in aggressive development and metastases of tumor combined with the mutagenic metabolic products and metabolic interactions^16^. Our study also demonstrated that longer tumor was related to inferior outcome, and was an independent risk factor for PFS but not for OS in oligometastatic EC patients. Although length of EC was not evaluated in the staging system, it has been proven to be a prognostic parameter for poor OS of lower stage EC (N0/N1), but not for N2/N3 EC. Moreover, tumor length was also regarded as a powerful marker for aggressiveness in ESCC^17,18^. The prognostic role of tumor length in PFS but not OS might be attributed to the diversity of tumor treatment after first-line therapy.

Sex, fistula and number of metastatic organs were also found to be independent prognostic factors for PFS of oligometastatic EC patients. Compared to male patients, female patients had a lower risk of death from oligometastatic EC, which was consistent with the results in metastatic EC patients^19^. The better outcome in female EC patients might be attributed to exposure to estrogens, which was found to inhibit squamous cell tumor growth^20^. Due to the development or treatment of EC, 17 patients experienced esophageal fistula in our study and had extremely poor prognosis with the median survival of 2–3 mo, on account of infection, hemorrhage or abscess followed by fistula^21^. A greater number of metastatic organs was related to worse prognosis. In spite of the indolent nature of oligometastatic EC, the large number of metastatic organs represents the higher growth potential and higher ability to metastasize, which could result in widespread metastasis and poor prognosis^22^.

Current guidelines recommended platinum-based chemotherapy for metastatic EC^23^. We found that additional radiotherapy for primary esophageal tumor was an independent protective factor for PFS and OS in oligometastatic EC. More importantly, local treatment for metastases was also associated with favorable prognosis in oligometastatic EC. Radical radiotherapy of all metastases has been demonstrated to achieve long-term survival in patients with oligometastatic breast cancer^24^ and lung cancer^25^. Patients with oligometastatic esophagogastric junction and gastric cancer were also found to obtain survival benefit from aggressive surgical treatment of metastases^26^. Our study is believed to be the first to demonstrate the efficacy of local treatment of metastases (whether partial or all metastases) in oligometastatic EC patients. Due to the lower invasiveness and limited development potential of oligometastatic EC, local radical treatment could further inhibit tumor development, invasion and migration. Therefore, on the basis of systemic chemotherapy, radical treatment of primary esophageal lesions and metastatic lesions may achieve long-term disease control, and even cure oligometastatic EC.

We further established visual nomogram models to quantitatively predict PFS and OS of oligometastatic EC patients, by incorporating the independent prognostic factors. The C-index of models remained at 0.739 and 0.696 after cross-validation for PFS and OS respectively, suggesting the powerful predictive capability of these models. Besides, DCA also demonstrated the favorable prediction of these nomogram models. The nomogram could provide the patient-specific estimates of OS and PFS at individual level using the patient’s characteristics. Aggressive local interventions are recommended for oligometastatic cancer patients, and patient-specific survival can be estimated using the nomogram. For example, if patients who have received aggressive local interventions but are still with high risk of progression and death, closer monitoring and further intensive treatment (like immunotherapy) may be considered. For patients who have not received prior local treatment and in the high risk of progression, further aggressive local interventions and intensive systemic treatment may be recommended. It is very interesting for further clinical studies to verify these hypotheses. We hope these predictive nomograms would offer feasible and practical reference for individualized management of EC patients in the near future.Our present study had some limitations. First, this was a retrospective study, and selection bias was inevitable. Prospective clinical trials for validation of our findings are needed in the future. Second, there was some heterogeneity in the treatment strategies and chemotherapeutic drugs among patients enrolled in our study. The optimal therapy patterns and the regimens also need to be determined in future studies.

## Conclusions

Oligometastatic EC patients with history of alcohol consumption, longer tumor had inferior PFS. And male patients with esophageal fistula, multiple metastatic organs were found to have inferior OS. Furthermore, local treatment for metastases and local radiotherapy for EC were demonstrated to be beneficial to the survival of oligometastatic EC patients. The prognostic nomograms were able to predict individual survival and provide evidence for clinical decision-making.

## Materials and methods

### Patient population and data collection

We have reviewed the medical records of metastatic EC patients in Shandong Cancer Hospital, between March 2013 and December 2018. The eligibility criteria for our retrospective analysis were as follows: (1) pathological diagnosis of EC; (2) newly diagnosed inoperable metastatic EC; (3) oligometastatic tumor that was defined as 1–5 metastases; and (4) available medical records. This study was performed in accordance with the principles of the 1975 Declaration of Helsinki and its later amendments or comparable ethical standards, and was approved by the Ethics Committee of Shandong Cancer Hospital. Due to the retrospective nature of the study, informed consent was waived by the Ethics Committee of Shandong Cancer Hospital and Institute.

The medical records, with regard to patient, tumor and treatment characteristics of each oligometastatic EC patient, were reviewed. Alcohol consumption was defined as the patients with a history of alcohol use. Longitudinal length was the length of EC which was measured under endoscopy, and analyzed as a continuous variable. And heavy smoking was defined as the smoking index ≥ 600. The tumor characteristics included the location, pathological type, cTNM stage based on the enhanced computed tomography (CT) scan, and characteristics of metastases. And treatment characteristics involved treatment protocol, local radiotherapy for EC and local treatment for oligometastases. The number of metastatic organs was defined as the number of organs which the metastases were located (including but not limited to metastatic lymph node, liver, lung and bone).

We applied a uniform database template to ensure consistent data collection. Tumor response was assessed by cervical, thoracic and abdominal enhanced CT, on the basis of Response Evaluation Criteria in Solid Tumors (RECIST) version 1.1. The survival data were collected from the Shandong Cancer Hospital medical record system or by telephone follow-up. PFS and OS were estimated from diagnosis of EC to the date of progression and the date of death or last follow-up, respectively.

### Statistical analysis

Univariate and multivariate Cox hazards models were used for OS and PFS analyses to assess the independent prognostic values for oligometastatic EC patients. Variables with *P* < 0.1 in univariate analyses were included in multivariate Cox models. The hazard ratio (HR) with 95% confidence interval (CI) were calculated, and *P* < 0.05 was considered to be statistically significant. Survival curves were depicted using the Kaplan–Meier method and compared with the log-rank test.

On the basis of results of multivariate Cox analyses, the nomogram was established for PFS and OS of EC patients. The final OS and PFS models were internally validated by bootstrapping using 100 resamples and cross-validation methods. Internal cross-validation with bootstrapping, which has been shown to be an effective method for creating unbiased patient-specific predictions, was used to validate the established nomograms^27^. The discriminatory ability and veracity of the Cox models was measured by C-index. And the calibration curve was generated by a plot of the predicted survival probabilities against the observed probabilities. Survival analyses were accomplished using SPSS version 24.0 (IBM Corp., Armonk, NY, USA), and nomogram was performed with the packages “rms” and “pec” in R version 3.6.0 (R Foundation for Statistical Computing, Vienna, Austria). DCA were performed to determine the clinical net benefit of different probability thresholds for the reliability of the model by R-script stdca. The statistical methods of this study were reviewed by Xiu-Bin Sun from the Department of Biostatistics, School of Public Health in Shandong University.

### Ethical approval and consent to participate

This study was approved by the Ethics Committee of Shandong Cancer Hospital and Institute. Due to the retrospective nature of the study, informed consent was waived by the Ethics Committee of Shandong Cancer Hospital and Institute.

## Data Availability

The datasets analysed during the current study are available from the corresponding author on reasonable request.
